# Editorial: Complementary therapies for neurological disorders: from bench to clinical practices

**DOI:** 10.3389/fneur.2023.1224144

**Published:** 2023-06-14

**Authors:** Yang Ye, Shuren Li, Lingyong Xiao, Jingling Chang

**Affiliations:** ^1^Department of Traditional Chinese Medicine, Peking University Third Hospital, Beijing, China; ^2^Department of Biomedical Imaging and Image-Guided Therapy, Medical University of Vienna, Vienna, Austria; ^3^Clinical Department of Acupuncture and Moxibustion, First Teaching Hospital of Tianjin University of Traditional Chinese Medicine, Tianjin, China; ^4^Department of Neurology, Dongzhimen Hospital, Beijing University of Chinese Medicine, Beijing, China

**Keywords:** neurological disorder, complementary therapy, stroke, Alzheimer's disease, herb medicine, acupuncture

Neurological disorders, such as stroke, Parkinson's disease (PD), dementia, and depression, impose heavy burdens on both individuals and society (1). A lot of attempts have been made to ease the symptoms of neurological disorders, but many challenges remain. Complementary therapies, such as acupuncture, moxibustion, herbal medicine, and Tai Chi have been widely used in the treatment of various neurological disorders worldwide, especially in China (2, 3). Numerous clinical trials and animal studies have been conducted to verify the efficacy of complementary therapies and explain their potential mechanisms (4, 5). However, the effectiveness and mechanisms of complementary therapies for treating neurological disorders remain controversial due to insufficient evidence. More well-designed clinical and basic studies in this field are urgently needed.

In order to provide a platform for authors in this field to share their latest research findings, we organized this Research Topic in *Frontiers in Neurology*, section Neurorehabilitation. A total of 53 manuscripts were received during the call for papers, 13 papers among them were finally accepted for publication following a rigorous peer-review process. The 13 accepted articles consist of five original research articles, one brief research report, two reviews, and five systematic review and meta-analyses. Many complementary therapies (e.g., acupuncture, electroacupuncture, and herbal medicine) and neurological disorders (e.g., stroke, depression, and PD) were involved in this Research Topic. These studies have well expanded our current knowledge about complementary therapies for neurological disorders.

## Original research article

PD, a progressive neurological disorder characterized by tremor, rigidity, and bradykinesia, is associated with the death of dopaminergic neurons in the brain (6). Jin et al. performed a pilot clinical trial involving 60 participants to evaluate the efficacy of Ukgansan, a traditional herbal formula composing seven plants, for improving clinical symptoms in patients with PD. This was a single-centered, randomized, controlled, assessor-blinded, pilot clinical trial conducted in South Korea. After 6 weeks of treatment, they found that additional use of Ukgansan improved the quality of life in PD patients with anxiety. Constipation is a common symptom in patients with PD, with a prevalence of 70–80% (7). Song et al. investigated whether electroacupuncture at “Tianshu” (ST25) promotes the restoration of the enteric nervous system and colonic motor function in a rat model of PD constipation. In that study, electroacupuncture at ST25 ameliorated abnormalities of the enteric nervous system and improved constipation symptoms in rats with PD constipation, possibly through maintaining the integrity of the colonic myenteric nervous plexus and regulating the neurotransmitters.

Stroke is a leading cause of death and disability in the world. Hemiplegia after stroke brings a serious burden on the economy and society (8). Lin et al. investigated the functional connectivity changes of cerebral hemispheres, trying to interpret the neural mechanism of scalp acupuncture on hemiplegia after stroke. This clinical study included 21 patients with hemiplegia after stroke and was performed in Beijing, China. They found that scalp acupuncture may promote rehabilitation in patients with hemiplegia after stroke. In addition, scalp acupuncture showed a bidirectional effect including strengthening functional connections of the bilateral motor cortex and weakening abnormal compensatory connections.

Autism spectrum disorder (ASD) is a common neurodevelopmental disorder in children characterized by deficits in social communication, narrow interests, and repetitive behaviors (9). Lee et al. observed the effectiveness and safety of a 6-month integrative treatment program (including herbal medicine, Floortime, and sensory enrichment therapy) on children with ASD in a prospective observational study. This study included 18 participants and was conducted in South Korea. Their results showed that the six-month integrative treatment program significantly relieved the symptoms of ASD children. This treatment program may be a potentially effective therapeutic strategy for ASD, but a placebo control group is needed in the future to further verify the efficacy.

Depression is the most prevalent psychiatric disorder characterized by depressed mood, social isolation, and anhedonia (10). Zhu et al. examined the anti-depressive effects of a traditional Chinese medicine decoction, Zi-Shui-Qing-Gan-Yin (ZSQGY), in a depressive animal model and in a cell model. Their results indicated that ZSQGY effectively improved depressive behavior in depressive animals. The mechanisms may be related to improvement in mitochondrion function, alleviation of neuroinflammation, and regulation of peroxisome proliferator-activated receptor-γ co-activator 1α.

## Brief research report

Equistasi^®^ is a vibrotactile device that has been used for rehabilitation in patients with movement disorders such as PD (11). Cruciani et al. briefly reported a pilot study conducted in Italy to explore the effect of Equistasi^®^ on somatosensory processing through the evaluation of high-frequency oscillations. They found that vibrotactile afference delivered by Equistasi^®^ could work through somatosensory processing, rather than by peripheral effects. Equistasi^®^ has the potential to restore equilibrium in disease states such as PD, but the efficacy needs to be further validated and the mechanism needs to be elucidated.

## Literature review

Ischemic stroke is a severe neurological disorder with a high mortality and disability rate (12). Acupuncture therapy has been widely used to treat ischemic stroke and the molecular mechanisms involved have been partly elucidated (13). Wang, Su et al. presented a comprehensive review on the mechanisms of acupuncture for enhancing cerebral perfusion in ischemic stroke. They concluded that acupuncture restores blood flow of ischemic tissue possibly via promoting hemodynamics and angiogenesis, releasing vasoactive substances, and improving microcirculation. Acupuncture has shown great potential to improve ischemic stroke in multiple ways and more high-quality clinical trials are needed to verify the efficacy of acupuncture. Cognitive impairment is another important condition that acupuncture has shown therapeutic potential (14). Zhou et al. performed a bibliometric review to explore the development context, research hotspots, and frontiers of acupuncture for cognitive impairment in the past three decades. This review stated that functional magnetic resonance imaging maybe better explain the therapeutic effect of acupuncture. In addition, they found that the effect of acupuncture on a single point is probably more convincing.

## Systematic review and meta-analysis

Five systematic review and meta-analyses were published in this Research Topic. Stroke and its complications are still hot topics for researchers. Wang, Chi et al. evaluated the evidence from current systematic reviews of acupuncture for early recovery after acute ischemic stroke. A total of seven systematic reviews including 114 randomized controlled trials (RCTs) were included and assessed in this study. Their results showed that acupuncture is a promising therapy that may improve the neurological function for patients recovering from acute ischemic stroke. However, the low quality of evidence affected the reliability of the results. Shoulder-hand syndrome is a common condition after stroke characterized by pain, hyperalgesia, swelling, and limited joint mobility (15). Feng et al. used Bayesian network meta-analysis to identify the most effective physical therapy for patients with poststroke shoulder-hand syndrome. A total of 45 RCTs were included in the final analysis. According to their results, electromyography biofeedback therapy combined with rehabilitation training is the best physiotherapy option, which could be used for patients with poststroke shoulder-hand syndrome to improve upper extremity motor function and relieve pain. Depression is another common condition affecting about one-third of stroke patients (16). Li et al. performed a systematic review and meta-analysis to assess the efficacy of Chinese herbal medicine (CHM) on poststroke depression in animal studies. A total of 14 studies with 12 CHMs were included. The results suggested that CHM could significantly improve depression-like behaviors and neurological function of poststroke depression animals.

Dysphagia is a common non-motor symptom in PD. Wu et al. assessed the efficacy of acupuncture on dysphagia in PD patients through meta-analysis method. Ten RCTs with 724 patients were included in this study. Their results showed that acupuncture may exert beneficial effects on dysphagia in PD, which supports acupuncture as an adjunctive treatment for dysphagia in patients with PD.

Diabetic peripheral neuropathy (DPN) is one of the most common complications of diabetes with a high rate of morbidity and mortality (17). Sun et al. systematically evaluated the efficacy of Tongmai Jiangtang Capsule (TJC) on diabetic peripheral neuropathy in patients. It included eight RCTs involving 656 participants for analysis. They found that TJC combined with conventional treatment significantly reduced the severity of DPN symptoms compared with conventional treatment alone. TJC is a promising drug for DPN based on the current evidence, more high-quality evidence is needed to validate the results.

In conclusion, this Research Topic shows the latest research advances both in animal and clinical studies in the complementary therapy field targeting neurological disorders. We believe that these studies will greatly expand our current understanding in this field and help us develop better therapies for patients with neurological disorders.

## Author contributions

All authors listed have made a substantial, direct, and intellectual contribution to the work and approved it for publication.
